# Potential synergistic effects of exercise and gut microbiota on post-stroke recovery: mechanistic insights and a proposed stage-specific research framework

**DOI:** 10.3389/fcimb.2026.1868705

**Published:** 2026-08-12

**Authors:** Luxing Zhou, Hongshuai Leng, Zhe Wang, Wenhong Liu

**Affiliations:** 1 Tianjin University of Sport, Tianjin, China; 2 Sports Injury and Rehabilitation Virtual Simulation Experiment Teaching Center, Tianjin University of Sport, Tianjin, China; 3 Department of Basic Teaching of Military Common Subjects, Logistics University of Chinese People’s Armed Police Force, Tianjin, China; 4 Department of Traditional Chinese Medicine Rehabilitation, Tianjin Rehabilitation and Convalescent Center of the People’s Liberation Army Joint Service Support Force, Tianjin, China

**Keywords:** exercise, microbiota–gut–brain axis, motor function, neuroplasticity, stroke rehabilitation

## Abstract

Post-stroke motor dysfunction imposes a profound burden on long-term quality of life. While conventional rehabilitation facilitates neuroplasticity, the marked interindividual variability in functional outcomes underscores the critical necessity for novel, synergistic adjuncts. Emerging evidence suggests that microbiota-related pathways may influence rehabilitation responsiveness; however, whether deliberate microbiota modification improves motor recovery remains uncertain. This narrative review summarizes evidence suggesting associations between post-stroke gut dysbiosis and motor outcomes, and integrates reported mechanistic pathways across barrier, immune, and metabolic domains to inform a phase-stratified exercise–microbiota framework for future trials. Stroke induces systemic disturbances that affect the intestinal barrier, immune regulation, and metabolic homeostasis. These perturbations are frequently accompanied by reduced abundance of short-chain fatty acid-producing taxa and altered neurotrophic signaling, changes that may constrain the neuroplastic milieu. Concurrently, structured exercise interventions have been demonstrated to remodel the gut ecosystem and modulate the systemic inflammatory milieu. In this context, microbiome-derived metabolites may function as signaling mediators that facilitate crosstalk among barrier integrity, immune regulation, and neural plasticity networks. Synthesizing these mechanistic insights, we propose a comprehensive framework that prioritizes the preservation of intestinal barrier integrity alongside titrated mobilization during the acute phase. Overall, combined interventions targeting exercise and the gut microbiota are biologically plausible and potentially feasible in stroke rehabilitation. While current evidence is promising, it remains nascent. Rigorous, phase-specific randomized controlled trials are imperative to confirm efficacy, define optimal dosing, and enable precision implementation.

## 1 Introduction

Stroke is a leading cause of long-term disability worldwide, and a large proportion of survivors experience persistent motor deficits (Li et al., 2024). Although conventional rehabilitation yields functional improvements, outcomes often remain suboptimal due to substantial interindividual variability in recovery trajectories, suggesting that multiple neuroplastic pathways and systemic constraints may limit recovery (Li et al., 2023).

Recent paradigm shifts suggest that exercise functions as a pleiotropic modulator, concurrently driving neural recovery and reshaping the gut ecosystem, including microbial composition and intestinal barrier integrity. Preclinical and early clinical studies indicate that it enriches beneficial taxa and enhances intestinal homeostasis, suggesting potential relevance for post-stroke recovery (Hill et al., 2023; Kofoed et al., 2024; Mackay et al., 2026). Accumulating evidence elucidates the complex interactions involving gut microbiota diversity, neuroinflammation, and functional recovery outcomes. These findings underscore the therapeutic potential of the microbiota–gut–brain axis (MGBA) in modulating neuroinflammation and enhancing functional recovery following stroke (Benakis et al., 2020; Feng et al., 2021).

Stroke triggers a time-dependent cascade in which early autonomic and neuroendocrine activation compromises gut barrier integrity and facilitates bacterial translocation (Wang et al., 2024). Beyond the first week, overt barrier disruption may attenuate, yet microbial community shifts and metabolite depletion can persist and sustain low-grade inflammation and metabolic constraints. Recognizing the temporal dynamics of stroke pathology facilitates distinction between an acute phase, marked by rapid intestinal barrier disruption, and a chronic phase characterized by sustained dysbiosis and metabolic imbalance (Granados-Martinez et al., 2024).

In this review, we synthesize evidence linking post-stroke dysbiosis to motor outcomes and summarize plausible mechanistic pathways across barrier, immune, and metabolic domains. We then propose a phase-stratified framework that aligns exercise prescription and microbiota-directed strategies with recovery biology and measurable biomarkers to support hypothesis-driven trial design.

### 1.1 Associations and candidate mechanisms linking gut dysbiosis to post-stroke motor recovery

**Figure 1:**
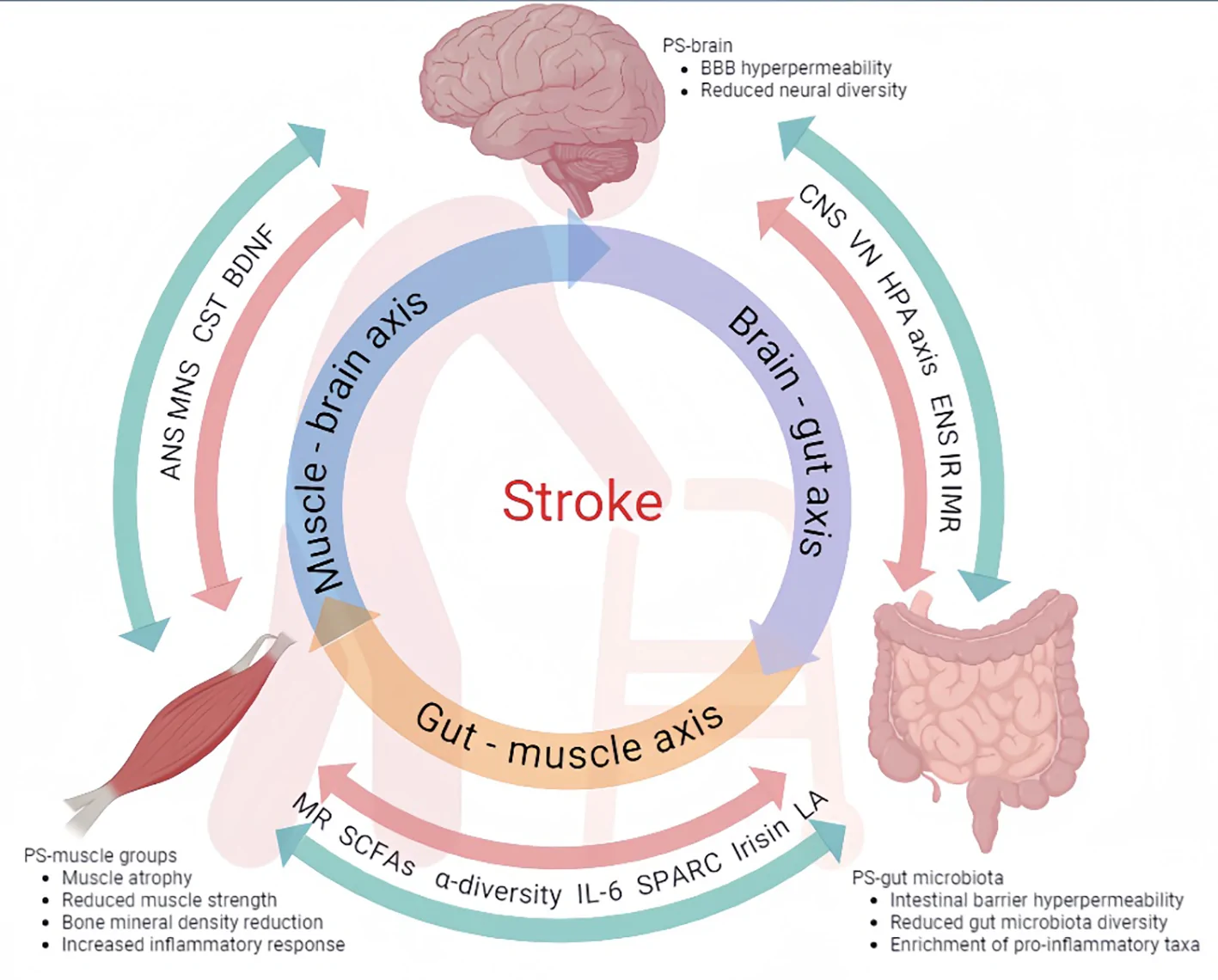
Interactions between the MGBA and exercise in post-stroke recovery. ANS, autonomic nervous system; MNS, mirror neuron system; CST, corticospinal tract; BDNF, brain-derived neurotrophic factor; CNS, central nervous system; VN, vagus nerve; HPA axis, hypothalamic–pituitary–adrenal axis; ENS, enteric nervous system; IR, inflammatory response; IMR, immune response; MR, metabolic response; LA, lactic acid; IL-6, interleukin-6. Created by the authors using BioRender.com.

As illustrated in **Figure 1**, targeted exercise-based interventions may mitigate gut dysbiosis via muscle–microbiome crosstalk, potentially helping to restore intestinal barrier integrity and to enhance neurotrophic signaling. Collectively, these mechanisms provide a rationale for investigating combined microbiota- and exercise-based interventions in stroke rehabilitation.

### 1.2 Stroke-induced gut dysbiosis

Stroke disrupts the gut microbial ecology and compromises barrier integrity, which are characterized by reduced microbial diversity and an increase in pro-inflammatory taxa. These changes evolve dynamically across acute and chronic phases. During the chronic or long-term period after cerebral infarction, gut-derived short-chain fatty acids (SCFAs) may remain reduced for months, accompanied by persistent gut dysbiosis (Martin et al., 2026). Clinically, depletion of SCFA–producing taxa and lower fecal SCFA levels are associated with more severe stroke deficits and a higher risk of unfavorable functional outcomes (Qu et al., 2024; Song et al., 2026). Because butyrate and other SCFAs can modulate neurotrophic signaling, including brain-derived neurotrophic factor (BDNF), as well as mitochondrial bioenergetics, sustained deficits may compromise neuroplastic support and hinder motor recovery (Lee et al., 2020; Sadler et al., 2020).

In the first week after stroke, sympathetic and neuroendocrine surges can precipitate rapid barrier collapse, increasing permeability and enabling microbial products and commensals to access sterile compartments, which aligns with early vulnerability to stroke-associated infections (Prame et al., 2025; Wang et al., 2024). After day 7, barrier measures may partially recover, yet dysbiosis can consolidate, with sustained loss of beneficial metabolite producers and persistent metabolite insufficiency that maintains low-grade neuroimmune activation (Cui et al., 2023; Prame et al., 2025).

Several studies have proposed composite dysbiosis metrics to summarize post-stroke microbiome disruption and reported associations with brain injury severity and clinical prognosis (Sun et al., 2021; Xia et al., 2019). For example, 18 genera differed between acute ischemic stroke patients and healthy controls, and more recent cohorts have also observed enrichment of *Lactobacillus* and *Streptococcus* in acute ischemic stroke (Xia et al., 2019; Yu et al., 2024); however, the causal and mechanistic contributions of these taxa remain incompletely defined. Post-stroke gut dysbiosis has been associated with intestinal mucosal inflammation, systemic inflammatory activation, and poorer functional outcomes. Experimental models suggest that increased barrier permeability, circulating inflammatory mediators, and gut-primed immune signaling may contribute to a neuroinflammatory milieu that constrains neuroplastic remodeling; however, these findings do not establish that microbiota modification improves motor recovery in patients (Iadecola et al., 2020). Across disease progression, this continuum—from gut barrier disruption and translocation of microbial products to reduced levels of microbiota-derived metabolites such as SCFAs—has been associated with poorer functional outcomes, supporting a plausible pathway through which dysbiosis may hinder motor recovery. These time-dependent changes suggest a shifting therapeutic focus—from early inflammation control to later metabolic and plasticity support. Early after stroke onset, evidence highlights rapid disruption of the intestinal mucosal barrier and heightened inflammatory activation, supporting a focus on barrier protection and inflammation control. In the subacute to convalescent stages after stroke, gut dysbiosis can persist, and long-term cerebral infarction models further show significant reductions in gut-derived SCFAs months after the event (Cui et al., 2023). These findings support prioritizing metabolic restoration and targeting neurotrophic and synaptic plasticity pathways in later-stage rehabilitation (Chen et al., 2019; Sadler et al., 2020; Wang et al., 2024).

#### 1.2.1 Impairment of intestinal barrier function

After stroke, circulating zonulin, a surrogate marker of intestinal barrier permeability, is elevated, and increased circulating levels of lipopolysaccharide (LPS) are consistent with microbial product translocation and systemic inflammation; both have been linked to greater stroke severity and poorer short-term functional outcomes (Zhu et al., 2023). Circulating LPS can engage Toll-like receptor 4 (TLR4) and trigger downstream signaling that culminates in nuclear factor kappa B (NF-κB) activation, thereby promoting pro-inflammatory gene expression and amplifying neuroinflammation after ischemic stroke (Xu et al., 2021).

Elevated zonulin, a marker of gut permeability, is linked to adverse outcomes, suggesting a shared pathophysiological pathway. In clinical stroke cohorts, higher circulating markers of gut barrier dysfunction or endotoxemia have been associated with poorer functional outcomes and increased short-term mortality, supporting the clinical relevance of restoring gut barrier integrity (Błaż et al., 2024). Notably, this dysbiotic state may impair responsiveness to exercise-based therapy by reducing metabolite availability and exacerbating systemic inflammation (Liu et al., 2020).

### 1.3 Mechanistic pathways linking gut microbiota dysbiosis to motor dysfunction via the metabolic–immune–neural axis

Post-stroke barrier disruption affects three layers: physical (mucus, tight junctions), chemical (antimicrobial, acidic environment), and immune (mucosal tolerance). Resulting endotoxemia promotes systemic inflammation, activates microglia, and disrupts vagal signaling, impairing motor plasticity. Key mechanisms include barrier dysfunction, metabolite deficits, and neuroimmune crosstalk—each representing a modifiable target.

The relative weight of these layers is phase-dependent, with barrier disruption and translocation dominating early, and metabolite-linked immunometabolic constraints becoming more prominent during subacute and chronic recovery (Prame et al., 2025).

#### 1.3.1 Mechanisms of bidirectional crosstalk between skeletal muscle and the gut microbiota

Skeletal muscle-to-gut signaling constitutes a pivotal arm of this feedback loop; specifically, exercise-induced myokines modulate microbial composition and function. During rehabilitation exercise, contracting skeletal muscle releases myokines and exerkines including interleukins and irisin that shape bile acid metabolism, immune tone, and intestinal microenvironment, thereby favoring taxa linked to SCFA production and lowering systemic inflammatory load (Saponaro et al., 2024).

Gut-derived metabolites not only serve as downstream indicators of microbial activity but also act as upstream modulators that influence training adaptability and physiological response to exercise. SCFAs support skeletal muscle mitochondrial biogenesis, insulin sensitivity, and fatigue resistance, which can influence exercise tolerance and the achievable training dose (Cavaliere et al., 2022). Experimental support for a mediating role of the microbiota comes from animal fecal microbiota transplantation studies in which exercise-associated microbiota transferred selected metabolic or neurogenic phenotypes to recipient animals. These model-specific findings do not establish microbiota-mediated effects in stroke survivors (Lai et al., 2018).

#### 1.3.2 Microbial metabolite deficiency and metabolic imbalance in stroke recovery

**Figure 2:**
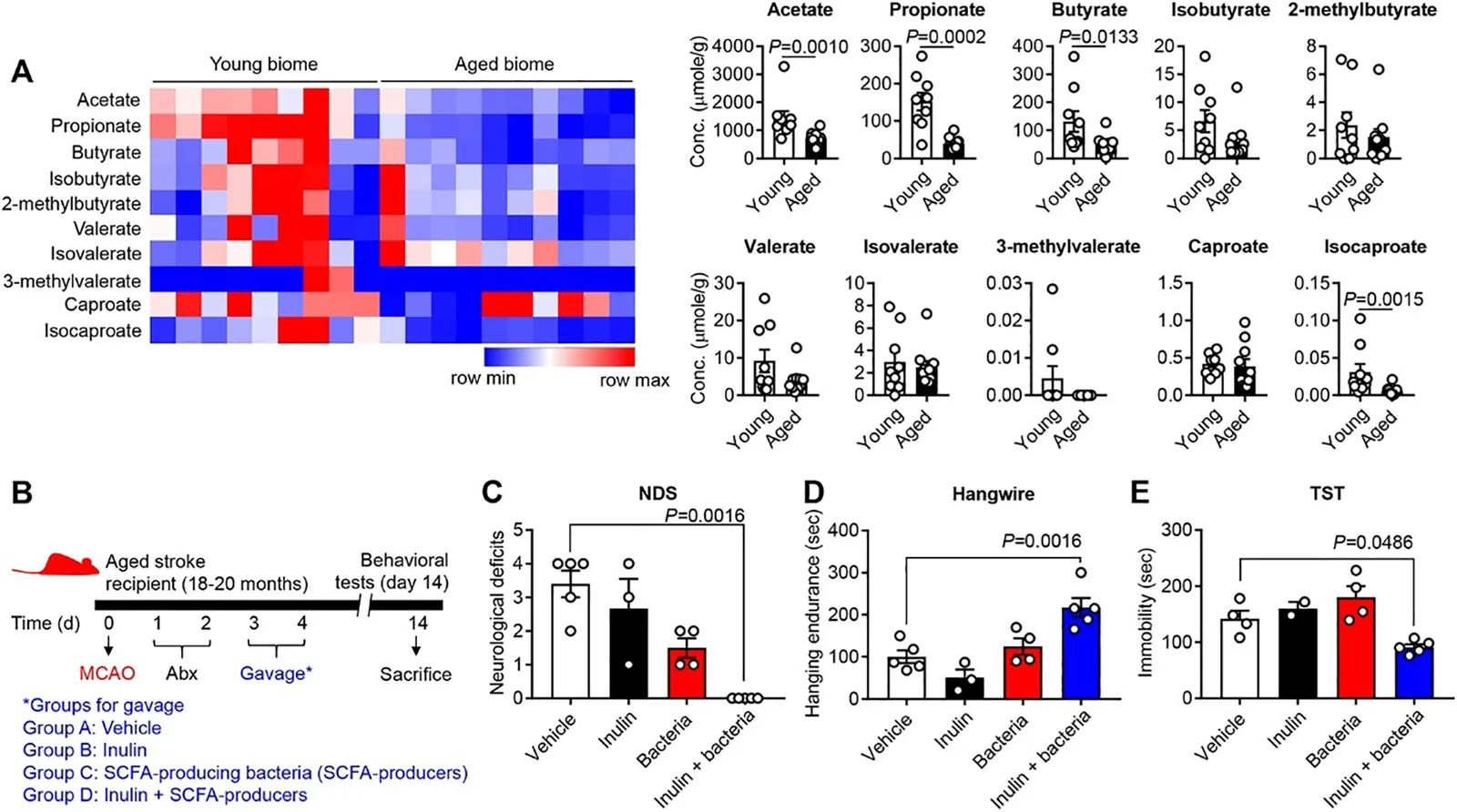
Fecal SCFA concentrations decrease with age, and combined treatment with SCFA-producing bacteria and the prebiotic inulin after stroke ameliorates behavioral disorders in aged mice. **(A)** Aging decreases the concentration of primary SCFAs in fecal samples of young (2–3 months) and aged (18–20 months) mice. Fecal samples were collected and analyzed using mass spectrometry. Graphs represent changes of individual SCFAs (young biome, n=9; aged biome, n=10). **(B)** To examine the effect of inulin and SCFA-producers on stroke outcome, four separate groups of mice were orally gavaged with 1) vehicle, 2) inulin, 3) SCFA-producers and 4) inulin+SCFA-producers. For SCFA-producers, the cocktail of *Bifidobacterium longum* (1×10^7^), *Clostridium symbiosum* (5×10^8^), Faecalibacterium prausnitzii (1×10^8^) and *Lactobacillus fermentum* (1×10^9^) were freshly cultured and gavaged to mice. Neurological deficit score (NDS) **(C)**, hangwire test **(D)** and TST **(E)** were performed at day 14 after MCAO (vehicle, n=5; inulin alone, n=3; SCFA-producers alone, n=4; inulin+SCFA-producers, n=5). For the TST, two mice from vehicle- and inulin-treated groups (one for each group) which showed spinning due to severe neurological deficits were excluded. Overall P values for NDS, hangwire test and TST was 0.0001, 0.0004 and 0.0036, respectively. Throughout, error bars represent mean±SEM. For group comparisons, Mann-Whitney U test (A) and ordinary one-way ANOVA or Kruskal-Wallis test (C, D and E) were used based on the normality of data assessed by the Shapiro-Wilk normality test, followed by Dunnett’s multiple comparison test. MCAO, middle cerebral artery occlusion; Abx, streptomycin treatment; NDS, neurological deficit score; TST, tail suspension test; SEM, standard error of the mean; Hangwire, hang-wire test used to assess motor coordination and grip strength. Reproduced from Lee et al., 2020 with permission. © 2020 American Heart Association, Inc.

Post-stroke dysbiosis is commonly accompanied by a reduction in SCFA availability, and the downstream consequences are mechanistically separable at the receptor and epigenetic levels (Ney et al., 2023). Butyrate can engage G protein-coupled receptor 109A (GPR109A) signaling in intestinal epithelial and immune compartments to support barrier integrity and constrain mucosal inflammation, thereby reducing systemic endotoxin pressure that can propagate neuroinflammation (Singh et al., 2014). Concurrently, butyrate acts as an endogenous histone deacetylase inhibitor, thereby promoting histone acetylation and upregulating BDNF transcription in brain regions governing learning-related plasticity (Intlekofer et al., 2013). Acetate and propionate can activate free fatty acid receptors 2 and 3 (FFAR2 and FFAR3) in enteroendocrine L cells and promote glucagon-like peptide-1 release, providing a plausible route by which microbial metabolites shape post-stroke metabolic resilience and training tolerance (Tolhurst et al., 2012). Consistent with this systems view, SCFA supplementation has been associated with improved blood–brain barrier (BBB) integrity and higher expression of tight junction proteins, including claudin-5 and occludin, in brain vasculature, thereby linking peripheral metabolite deficits to a neuroplasticity-limiting inflammatory milieu (Hoyles et al., 2018). **Figure 2** illustrates behavioral improvement after SCFA and inulin co-supplementation in stroke models (Lee et al., 2020).

#### 1.3.3 Tryptophan metabolism along the microbiota–gut–brain axis in stroke

**Figure 3:**
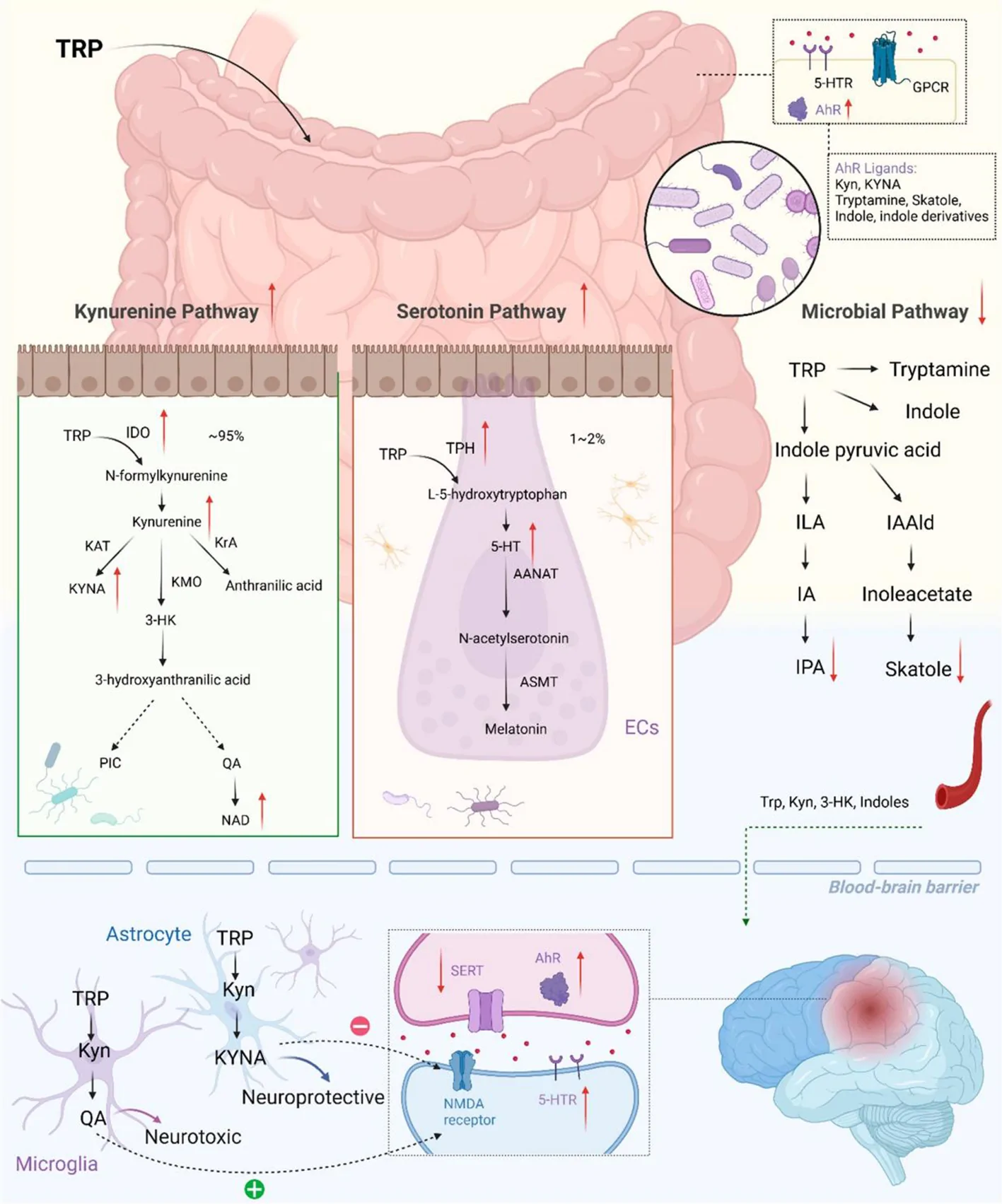
Tryptophan metabolism along the microbiota–gut–brain axis and its main alteration in stroke. The schematic depicts the three major Trp branches—kynurenine, serotonin, and indole derivatives—with stroke-associated shifts (e.g., IDO1/TDO-mediated diversion toward Kyn, serotonergic perturbations, and altered indole–AhR signaling) that converge on barrier integrity, neuroinflammation, and synaptic plasticity relevant to motor recovery. Reproduced from (Shen and Mu, 2024), under the Creative Commons Attribution 4.0 International License (CC BY 4.0). ^©^ 2024 The Authors.

The principal host and microbial tryptophan pathways and their stroke-associated alterations are summarized in **Figure 3**. The gut microbiota may modulate post-stroke neuroplasticity, while exercise reprograms peripheral kynurenine handling in skeletal muscle to reduce neurotoxic pressure on post-stroke circuit remodeling (Sadler et al., 2020).

Tryptophan metabolism has emerged as a key component of the microbiota–gut–brain axis in stroke, spanning host kynurenine metabolism and microbe-derived indole pathways that shape systemic immunity and neuroinflammatory signaling after ischemia (Shen and Mu, 2024). Clinical and mechanistic studies indicate that ischemic stroke is accompanied by dysregulated tryptophan catabolism, including shifts in kynurenine-pathway balance toward neurotoxicity, often reflected by an increased quinolinic acid to kynurenic acid ratio, together with disturbances in gut-derived indole metabolites such as indole-3-propionic acid that may impair gut–brain signaling (Gold et al., 2011). In acute stroke cohorts, higher circulating kynurenine and markers of kynurenine-pathway activation have been linked to adverse immune complications and worse outcomes, supporting a connection between tryptophan metabolism and post-stroke neuroimmune states (Dylla et al., 2025). Under post-stroke inflammatory conditions, elevated levels of pro-inflammatory cytokines, particularly interferon-γ (IFN-γ), strongly induce the expression of indoleamine 2,3-dioxygenase (IDO) (Lovelace et al., 2017). This enzymatic upregulation diverts tryptophan metabolism from serotonin synthesis toward the kynurenine pathway, precipitating the accumulation of neurotoxic kynurenine (KYN). Circulating KYN readily crosses the BBB and is metabolized by activated microglia into quinolinic acid, a potent N-methyl-D-aspartate receptor agonist that mediates excitotoxicity and neuronal death (Fukui et al., 1991).

Exercise training activates the skeletal muscle peroxisome proliferator-activated receptor-γ coactivator 1α (PGC-1α) pathway and increases the expression of kynurenine aminotransferases that divert circulating kynurenine toward kynurenic acid in the periphery. Because kynurenic acid does not readily cross the BBB, this peripheral shunting reduces kynurenine availability for central conversion into quinolinic acid and thereby lowers excitotoxic constraint on post-stroke neuroplasticity.

Tryptophan metabolites derived from the gut microbiota function as critical regulatory nodes that enhance epithelial barrier integrity and promote immune homeostasis. Indole derivatives act as ligands for the aryl hydrocarbon receptor (AhR) in epithelial and innate lymphoid compartments, promoting barrier-protective programs including interleukin-22-associated responses and thereby reducing translocation-driven inflammatory cues that sustain IDO1 induction and kynurenine pathway flux (Ye et al., 2022; Yoo and Oh, 2023).

Together, this gut supply, inflammation-driven shunting, and muscle diversion model provides a concise biochemical rationale for combining microbiota-targeted strategies with exercise-based rehabilitation to enhance the permissive milieu for motor network reorganization while reducing excitotoxic and inflammatory burden (Martin et al., 2020; Stone et al., 2022).

Stroke-associated inflammation can increase IDO activity and elevate the kynurenine-to-tryptophan ratio, favoring downstream metabolites that amplify excitotoxic and microglia-driven neuroinflammation. This is directly relevant to motor cortical remodeling because plasticity in the primary motor cortex (M1) depends on a tightly regulated excitatory–inhibitory balance and neurotrophin signaling. Exercise may shift this axis via skeletal-muscle PGC-1α–linked induction of kynurenine aminotransferases, promoting peripheral conversion toward kynurenic acid and reducing circulating kynurenines available to the brain. In parallel, stroke-induced loss of microbiota-derived indole AhR ligands may bias AhR signaling toward host-derived kynurenine ligands, providing an additional rationale for microbiota strategies that restore indole production during rehabilitation (Chen et al., 2025).

Stroke-induced dysbiosis initiates a cascade involving barrier dysfunction and reduced microbial metabolite availability, which together enhance systemic inflammation and constrain neuroplastic remodeling relevant to motor recovery. Exercise and microbiota-directed strategies converge on this cascade by reducing inflammatory induction and increasing peripheral kynurenine diversion toward kynurenic acid, strengthening the biochemical permissiveness for rehabilitation-induced circuit rewiring (Martin et al., 2020; Seo and Kwon, 2023).

#### 1.3.4 Disruption of immune homeostasis and neuroinflammatory processes

Disruption of intestinal immune homeostasis after stroke is reinforced by microbiota dysbiosis and weakened mucosal defenses (Shen et al., 2025). This disruption promotes translocation of pathogen-associated molecular patterns such as lipopolysaccharide into the lamina propria, activating dendritic cells and downstream pro-inflammatory cascades (Galea, 2021). Concurrently, depletion of beneficial commensals, particularly taxa that support short-chain fatty acid availability, may limit regulatory T cell differentiation and reduce interleukin 10 production, thereby weakening intestinal immune homeostasis (Atarashi et al., 2011). Such immune imbalance may favor activation of the NLR family pyrin domain-containing 3 inflammasome activation and downstream interleukin 1 beta and interleukin 18 signaling, amplifying mucosal inflammation and potentially disrupting tolerance mechanisms (Seo et al., 2015).

#### 1.3.5 Vagal pathway disruption in the microbiota–gut–brain axis after stroke

The microbiota–gut–brain axis is a bidirectional signaling network that links intestinal microbes to central nervous system function through microbial metabolites, mucosal immune signaling, and neuroendocrine pathways, with vagal afferents providing a rapid interoceptive channel. Experimental evidence indicates that select microbiota-regulated luminal metabolites can directly modulate vagal afferent activity via receptor-mediated signaling, offering a mechanistic substrate for microbiome-dependent control of neuromodulatory drive during rehabilitation training (Jameson et al., 2025). After stroke, dysbiosis and barrier disruption can reduce beneficial metabolites and perturb enteroendocrine and enterochromaffin signaling, which plausibly blunts vagal afferent firing patterns and lowers the gain for skill acquisition and retention (Prame et al., 2025). Clinically, rehabilitation paired with implantable vagus nerve stimulation improved upper-limb impairment in a pivotal randomized, blinded device trial involving patients with chronic ischemic stroke (Dawson et al., 2021). Based on these findings, the paired vagus nerve stimulation (VNS) system has received Food and Drug Administration approval for the treatment of upper limb motor deficits (Dawson et al., 2016). Mechanistically, outcome locked vagus nerve stimulation drives selective circuit modulation through cholinergic reinforcement and accelerates consolidation of skilled motor behavior, directly linking vagal traffic to M1 plasticity relevant for recovery (Bowles et al., 2022). Taken together, vagal signaling should be considered a motor-learning relevant interface rather than only an inflammatory reflex, and it provides a biologically plausible convergence point for microbiota-targeted strategies and task-specific training.

## 2 Microbiome-based therapeutic strategies in stroke rehabilitation

Probiotics and prebiotics represent promising therapeutic modalities for recalibrating the post-stroke gut microbiome. Preclinical evidence suggests they can enhance barrier integrity, modulate immune tone, and increase BDNF (Wang et al., 2023). Their therapeutic effects may differ by recovery phase, targeting barrier stabilization in the acute phase and metabolic or plasticity support in the chronic phase. In the acute phase, they may attenuate neuromuscular atrophy and prevent secondary neuronal injury due to disuse. In the chronic or late post-stroke stage, motor gains from conventional rehabilitation often slow, consistent with a reduced window of heightened neuroplasticity (Ballester et al., 2019). Microbiota-targeted approaches, including strategies that restore microbiota-derived metabolites such as SCFAs, may complement rehabilitation by modulating metabolic homeostasis and inflammation with potential benefits for circuit plasticity and recovery (Sadler et al., 2020).

### 2.1 Exercise–microbiota interaction in post-stroke rehabilitation

Exercise modulates the gut–muscle–brain axis through reciprocal interactions between contracting skeletal muscle and the gut microbiome (Allen et al., 2018). In human studies, structured exercise training improves metabolic and immune homeostasis and has been associated with stronger gut barrier function and lower endotoxemia or inflammatory markers (Varghese et al., 2024). In preclinical models, exercise reproducibly increases microbial diversity and enriches SCFA-producing taxa, with corresponding increases in SCFAs (Motiani et al., 2020). Exercise-induced muscle signals, including myokines such as irisin, can engage BDNF-related pathways, and aerobic training can increase circulating BDNF, supporting neuroplasticity (Wrann et al., 2013).

We propose that regular exercise and microbiota-directed strategies be investigated as potentially complementary interventions rather than assuming a clinically established synergistic effect. Muscle contraction releases myokines and myometabolites that can reinforce intestinal barrier function, reshape bile acid signaling, and select for SCFA-producing taxa, while the remodeled gut metabolome feeds back to muscle and immune compartments to dampen endotoxemia and systemic inflammation (Steensberg et al., 2000). This bidirectional muscle–gut loop reduces microglial priming and creates a permissive milieu for use-dependent plasticity in the motor network, thereby providing a mechanistic rationale for integrating exercise dosing with microbiome modulation in post-stroke rehabilitation (Mancin et al., 2023).

Exercise-induced myokines facilitate the enrichment of SCFA-producing taxa such as *Roseburia* and *Bifidobacterium*, which contribute to improved metabolic, immune, and intestinal barrier homeostasis (Steensberg et al., 2000). This cascade enhances mucosal barrier function, promotes glucose uptake, boosts mitochondrial oxidative capacity, and inhibits histone deacetylase (HDAC) activity, collectively supporting neuromuscular and neuroplastic resilience and neuroprotective metabolic optimization (Zhu et al., 2025). Myokines such as IL-6 can activate epithelial AMP-activated protein kinase (AMPK) signaling, leading to enhanced expression and assembly of tight junction proteins and thereby strengthening intestinal barrier function (Chae et al., 2023). Among exercise-induced factors, fibroblast growth factor-2 (FGF-2)—although not a classical myokine—has been shown to inhibit NF-κB phosphorylation in macrophages and reduce pro-inflammatory cytokine secretion, thereby contributing to an anti-inflammatory systemic milieu (Pan et al., 2020). Exercise-induced shifts in the gut microbiota can enrich SCFA-producing taxa, increase SCFA availability, and support barrier function while lowering systemic inflammatory tone (Allen et al., 2018). Preclinical evidence, including stroke models, suggests that exercise can dampen endotoxin-responsive inflammatory signaling, including TLR4 and NF-κB pathways, thereby attenuating neuroinflammation (Ma et al., 2013). At the neural remodeling level, aerobic exercise can increase circulating brain-derived neurotrophic factor and enhance neuroplasticity-related measures in people with stroke, supporting motor relearning (Mackay et al., 2026). Microbiota-derived SCFAs may influence neuroplasticity via vagal afferent pathways and molecular mechanisms associated with BDNF expression and synaptic remodeling (O'Riordan et al., 2022).

**Figure 4:**
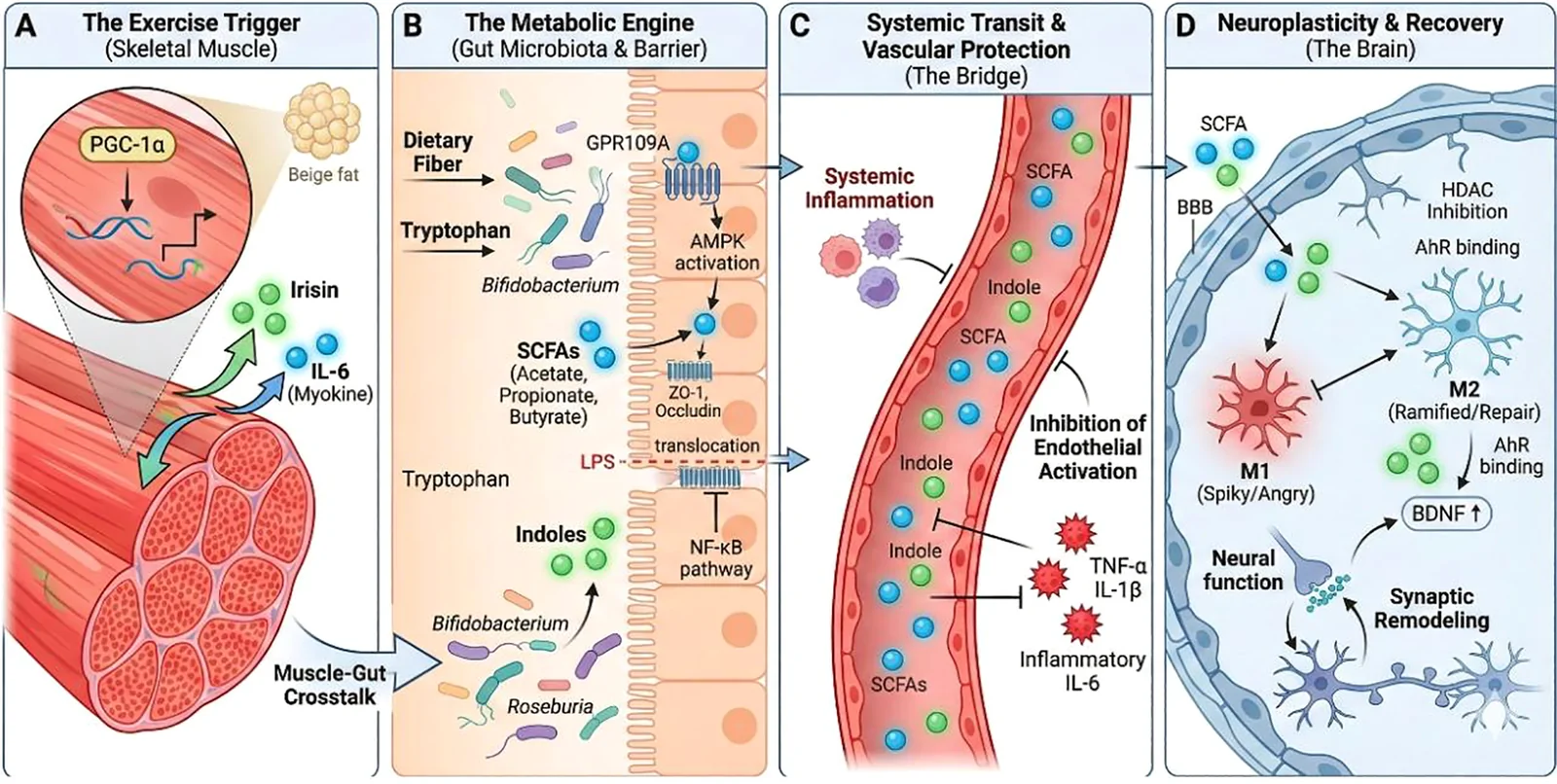
Proposed exercise-modulated muscle-gut-brain axis in post-stroke recovery. **(A)** Exercise activates skeletal muscle PGC-1α signaling and induces the release of myokines, including irisin and IL-6. **(B)** Microbial metabolism of dietary fiber and tryptophan generates SCFAs and indole metabolites, which may engage epithelial signaling pathways and support intestinal barrier integrity. **(C)** These metabolites enter the systemic circulation and may attenuate systemic inflammation and endothelial activation. **(D)** SCFAs may cross the BBB and inhibit HDAC activity, whereas indole metabolites may engage AhR signaling. Together, these mechanisms may shift microglia toward reparative-like states, increase BDNF expression, and support synaptic remodeling and neural recovery. PGC-1α, peroxisome proliferator-activated receptor gamma coactivator 1-alpha; IL-6, interleukin-6; SCFAs, short-chain fatty acids; GPR109A, G protein-coupled receptor 109A; AMPK, AMP-activated protein kinase; LPS, lipopolysaccharide; NF-κB, nuclear factor kappa B; TNF-α, tumor necrosis factor alpha; IL-1β, interleukin-1 beta; BBB, blood-brain barrier; HDAC, histone deacetylase; AhR, aryl hydrocarbon receptor; BDNF, brain-derived neurotrophic factor. In this figure, M1 and M2 refer to pro-inflammatory-like and reparative-like microglial states, respectively. Created by the authors using BioRender.com.

As illustrated in **Figure 4**, proposed muscle–gut–brain interactions may link peripheral metabolic and immune signals with neuroplasticity-related pathways relevant to post-stroke recovery. Skeletal muscle contraction stimulates PGC-1α-dependent pathways to release myokines such as irisin and interleukin-6, which facilitate muscle-gut crosstalk and enrich short-chain fatty acid-producing taxa including *Bifidobacterium* and *Roseburia*. These microbial metabolites subsequently activate AMPK signaling to reinforce intestinal tight junction proteins while inhibiting NF-κB pathways to prevent lipopolysaccharide translocation and systemic inflammation. Upon crossing the blood-brain barrier, circulating acetate, butyrate, and indole derivatives exert neuroprotective effects by inhibiting histone deacetylases and binding aryl hydrocarbon receptors to shift microglial polarization toward a reparative M2 phenotype. This metabolic cascade ultimately upregulates brain-derived neurotrophic factor expression and fosters a permissive microenvironment for synaptic remodeling and functional motor recovery.

Across the recovery trajectory, aerobic and resistance training exert phase-specific effects on the microbiome. Reports suggest that resistance training can attenuate TLR4/NF-κB signaling and reduce inflammatory tone, whereas combining aerobic exercise with prebiotic or probiotic support may enrich SCFA-producing taxa and affect mitochondrial efficiency and coordination; whether such effects differ by recovery stage remains to be determined. In preclinical models, running increased synaptic density in the M1, an effect amplified by butyrate co-supplementation (Chen et al., 2024). Limited human evidence suggests that structured exercise programs may shift the gut microbiome toward increased representation of SCFA-producing taxa, although findings vary across cohorts and study designs (Pérez-Prieto et al., 2024).

Both aerobic and resistance exercise training can reduce systemic inflammatory markers and may improve biomarkers of gut barrier integrity, although the direction and magnitude of change appear to depend on training dose, intensity, and context (Khalafi et al., 2023). Resistance training activates skeletal muscle mTORC1 signaling and supports anabolic and metabolic remodeling, and human studies and recent syntheses suggest that resistance training can modestly shift the gut microbiome toward a greater representation of butyrate-producing taxa, consistent with an SCFA-oriented metabolic profile (Levitt et al., 2022). However, some studies have reported that resistance training induces gut microbiota changes comparable in direction—but not necessarily in magnitude—to those observed with aerobic training. These findings may be influenced by differences in intervention duration, intensity, or cohort characteristics, warranting further standardized comparisons (Boytar et al., 2023). Discrepancies in microbiota composition and functional outcomes across existing studies may be attributable to methodological heterogeneity—particularly in sample size, exercise intensity, and intervention duration (Boytar et al., 2023). Standardizing these variables in future trials will be essential for elucidating dose–response relationships and identifying optimal exercise–microbiota pairings (Boytar et al., 2023). The gut microbiota may require sustained or higher-intensity exercise stimuli to elicit significant compositional shifts, as short-term training is often inadequate to overcome its inherent ecological stability and induce beneficial remodeling (Chavez-Arroyo et al., 2025). Therefore, developing individualized, evidence-based exercise programs that account for functional capacity and onset timing warrants evaluation in prospective studies.

Both aerobic and resistance exercise training are recommended components of post-stroke rehabilitation. Aerobic training supports cardiorespiratory fitness and cardiometabolic risk management, whereas resistance training targets strength and helps preserve skeletal muscle mass, which may reduce post-stroke deconditioning and sarcopenia-related functional decline (Billinger et al., 2014). Early initiation of structured exercise after stroke should be individualized and titrated to medical stability and patient tolerance, as very early high-dose mobilization has shown mixed effects in randomized trials (Dromerick et al., 2021). Rehabilitation delivered within an optimal post-stroke window may better leverage heightened neuroplasticity, and combining exercise with microbiota-oriented strategies has been proposed as a timing-sensitive approach to support recovery biology (Dromerick et al., 2021).

### 2.2 Microbiota-related pathways, neuroplasticity, and motor recovery after stroke

Human observational studies have associated post-stroke dysbiosis with greater neurological impairment and poorer functional outcomes, whereas animal experiments indicate that microbiota manipulation can modify neuroinflammatory and behavioral responses within specific experimental models (Singh et al., 2016; Sun et al., 2021). These findings support biological plausibility but do not establish that modifying the gut microbiota will improve motor recovery in patients.

A two-sample Mendelian randomization analysis identified suggestive associations between genetically proxied bacterial taxa and post-stroke outcomes. Genetically proxied greater abundance of the family Oxalobacteraceae and the genus Ruminococcaceae UCG014 was suggestively associated with better motor recovery, whereas Enterobacteriales and Enterobacteriaceae were associated with motor weakness. However, none of these findings remained significant after Bonferroni correction. Mendelian randomization estimates depend on instrumental-variable assumptions and reflect genetically proxied microbial traits; they do not directly estimate the effects of modifying these taxa through probiotics, diet, fecal microbiota transplantation, or exercise. These findings should therefore be considered hypothesis-generating and require confirmation in prospective interventional trials (Qu et al., 2024; Sanderson et al., 2022). Exploratory enrichment analyses implicated pathways related to synaptic regulation, muscle-cell cytoskeletal organization, and small-molecule transport, but these pathway-level findings remain indirect and require experimental validation.

Systemic inflammation triggered by gut barrier leak is not confined to peripheral organs. After stroke, lipopolysaccharide can enter the circulation and amplify inflammatory signaling that primes microglia toward a pro-inflammatory state. This state is linked to exaggerated synaptic elimination through complement-associated programs and to impaired neurotrophin signaling including reduced BDNF expression, which together constrains experience-dependent remodeling of peri-infarct motor networks. Consequently, restoring barrier integrity and immunometabolic homeostasis alleviates the inflammatory braking on neuroplasticity, thereby creating a permissive milieu for circuit reorganization (Xie et al., 2025).

Neuroplasticity represents the fundamental basis for motor function recovery following stroke. Impaired neurogenesis results from a reduction in the number and/or functional capacity of neural stem and progenitor cells (Walgrave et al., 2021). In post-stroke patients, microbiota dysbiosis leads to depletion of SCFAs, which intensifies NF-κB pathway activation and oxidative stress, compromises BBB integrity, and exacerbates neuroinflammation. Maintenance of gut microbiota homeostasis supports neurogenesis and synaptic remodeling via coordinated metabolic–immune–neural signaling cascades, including SCFA-mediated HDAC inhibition and neurotrophin regulation. Preclinical studies suggest that SCFA-related signaling may support neurotrophin expression and synaptic remodeling through HDAC-dependent and immune-mediated pathways (Intlekofer et al., 2013; Sadler et al., 2020). Evidence from animal models indicates that probiotic administration can restructure gut microbial composition, attenuate neuroinflammatory cascades, and enhance neurological functional outcomes in post-stroke settings (Holcomb et al., 2025). Beyond their anti-inflammatory effects, SCFAs inhibit HDAC activity activity and promote the biosynthesis of serotonin precursors, thereby supporting neurotransmission and mood regulation. These mechanisms support dopaminergic neurotransmission in the basal ganglia, thereby contributing to improved motor coordination and functional recovery. Probiotics can restore SCFA levels, suppress pro-inflammatory cytokines such as tumor necrosis factor-α, and help maintain mitochondrial function and neuronal homeostasis (Baba et al., 2025). Furthermore, microbial metabolites derived from select bacterial strains have been shown to improve exercise endurance by modulating energy metabolism and redox balance through the gut–liver–brain axis (Morita et al., 2023). Outside stroke-specific settings, *Bacteroides uniformis* and its preferred substrate have been associated with improved endurance-related metabolic performance in mice and human males (Morita et al., 2023). These findings may inform hypotheses regarding rehabilitation tolerance, but they do not demonstrate improved synaptic plasticity or post-stroke motor recovery. These findings suggest that enhanced microbial engraftment may potentiate the neurofunctional benefits of targeted microbiota interventions (Picca et al., 2018). Beyond microbiome-derived metabolites, serotonergic tone can influence plasticity; however, pharmacologic modulation lies outside the scope of this microbiome-centered framework. Here we focus on tractable microbial and metabolite targets that interface directly with barrier, immune, and neural domains relevant to motor recovery (Melo et al., 2024).

#### 2.2.1 Gut microbiota regulation of skeletal muscle function in stroke

Microbiota-derived metabolites and immune or vagal signaling may influence neural and neuromuscular processes relevant to post-stroke recovery (Ohara and Hsiao, 2025). In the Mendelian randomization analysis by Qu et al., genetically proxied Peptostreptococcaceae, Lachnospiraceae NK4A136 group, and Lachnospiraceae UCG004 showed suggestive associations with favorable overall functional outcomes rather than specifically measured motor recovery. None of these associations survived correction for multiple testing; therefore, these taxa should be considered candidate genetic signals rather than established determinants of recovery or therapeutic targets (Qu et al., 2024).

SCFAs may cross the BBB and act via HDAC inhibition or GPRs to enhance synaptic plasticity and neuromuscular function (Frost et al., 2014). Preclinical studies indicate that *Bacteroides uniformis* may enhance exercise endurance through a gut-liver metabolic axis (Morita et al., 2023). Specifically, acetate and propionate derived from this strain have been shown to upregulate hepatic gluconeogenic genes, such as *Pck1*. This metabolic adaptation helps maintain glucose homeostasis during sustained physical activity, thereby potentially supporting the high metabolic demands of intensive rehabilitation training (Morita et al., 2023; Qu et al., 2024).

In the same Mendelian randomization analysis, genetically proxied Enterobacteriales and Enterobacteriaceae were suggestively associated with motor weakness. However, these findings were not significant after Bonferroni correction and do not establish that reducing the abundance of these taxa would improve motor function (Qu et al., 2024). Separately, one recent three-arm randomized trial reported greater short-term improvements in physical and motor outcomes with combined exercise and probiotic supplementation than with either intervention alone. However, this single study does not establish a general class effect or confirm that the observed benefit was mediated by microbiota modification (Ademoyegun et al., 2025).

Accordingly, this review distinguishes associations identified in clinical observational studies, genetically proxied associations estimated by Mendelian randomization, effects observed after controlled experimental manipulation within specific animal models, and treatment effects evaluated in randomized clinical trials. These evidence types address different questions and should not be interpreted interchangeably. In particular, Mendelian randomization findings do not substitute for clinical trials that directly test whether microbiota-targeted interventions improve post-stroke recovery.

## 3 Potential complementarity of exercise and microbiota-directed interventions in functional recovery

**Table 1** summarizes clinical studies in which microbiota-targeted adjuncts were added to usual care or routine rehabilitation without a prespecified structured exercise program. These studies provide preliminary evidence regarding the feasibility and potential effects of microbiota-directed adjuncts, but they do not demonstrate an interaction or synergistic effect between exercise and microbiota modification. A hypothesized complementarity is that microbiota-directed interventions may reduce systemic inflammatory constraints, whereas exercise provides activity-dependent input for neural remodeling. This hypothesis requires direct evaluation using factorial or other interaction-focused clinical trial designs.

**Table 1:** Representative clinical studies of microbiome-targeted adjuncts in stroke rehabilitation.

Study, design	Population and analyzed sample; group-specific age (years)	Intervention	Comparator	Timing/ duration (w)	Outcomes assessed	Motor-specific outcome
(Wang et al., 2023); RCT (parallel-group)	400 (200/200); 63.66 ± 6.75/63.60 ± 8.49	Combined live tablets of *Bifidobacterium*, *Lactobacillus*, *Enterococcus*, *Bacillus cereus*; oral, 3 tablets tid × 2 w + conventional therapy	Conventional therapy alone	Acute start; 2	I↑, V↑, VI↓	NA
(Rahimi et al., 2024); RCT (single-center ICU)	120 randomized (60/60); 111 analyzed (56/55); 60.64 ± 11.85/59.64 ± 8.39	Postbiotic 2000 mg qd (SCFAs + exopolysaccharides of *Lactobacillus paracasei*), feeding-tube; plus standard care	Maltodextrin placebo qd via feeding-tube; standard care	Acute; 1	I↔, II↔, VI↓	NA
(Xin et al., 2024); Retrospective study	153 (80/73); 72.29 ± 2.20/72.74 ± 3.06	*Bifidobacterium bifidum* triplex live-bacteria preparation 0.42 g tid, oral × 4 w	Standard care (incl. rehab) without probiotic	Chronic; 4	II↑, V↑, VI↓	↑
(Arslan et al., 2025); Prospective study	72 (36/36); 71.25 ± 14.38/71.17 ± 13.93	*Saccharomyces boulardii* 250 mg, bid × 7 d; oral or nasogastric administration; plus standard care	Standard care without probiotics	Acute start; intervention, 1; observation/outcome assessment, 4	II↑, VI↓	NA

RCT, randomized controlled trial; ICU, intensive care unit; CG, control group; IG, intervention group; y, years; incl., including; d, day; w, week; D, postoperative day; qd, once daily; bid, twice daily; tid, three times daily; Arrows: ↑ improved; ↓ decreased. Outcome codes: I, gut microbiota composition and SCFA profile; II, neurological function; III, cerebral infarct volume/surrogates; IV, visceral function; V, depressive/affective symptoms; VI, neuroinflammation indices. NA, not assessed as a motor-specific outcome; ↔, no statistically significant between-group difference.

Convergent patterns emerged across domains. Adjunctive probiotics/postbiotics consistently improved inflammation-related readouts and gut microbiota and SCFA profiles, with several trials also reporting gains in global neurological function and affective symptoms (Rahimi et al., 2024; Wang et al., 2023). Notably, formal motor scales were rarely captured or were not reported, underscoring a persistent evidence gap at the level of rehabilitation-salient motor endpoints. Collectively, these findings underscore both the biological plausibility and clinical feasibility of microbiome-targeted adjuncts in stroke rehabilitation. However, future trials should rigorously predefine motor function as a primary endpoint and adopt factorial designs to systematically evaluate the synergistic effects of combined exercise and microbiome modulation.

Recent clinical evidence is emerging but remains formulation-specific. Available studies include a randomized intensive care unit trial using a postbiotic strategy, multi-strain live formulations, and single-species supplementation, with reported benefits spanning neurological severity, infection-related outcomes, and selected functional endpoints. However, substantial heterogeneity in strain specificity, dosage regimens, and delivery matrices precludes robust cross-study generalization and underscores that efficacy should be interpreted at the strain or product level with explicit reporting rather than as a class effect of probiotics (Rahimi et al., 2024).

Preclinical models, despite heterogeneity, support microbiota interventions via barrier repair, SCFA restoration, and reduced neuroinflammation (**Table 2**). In these preclinical models, treatment groups were compared against vehicle-treated, saline-treated, or sham-operated controls, depending on the specific experimental design.

**Table 2:** Preclinical stroke models of microbiome-targeted therapies: intervention features, dosing/timing, and multi-domain outcomes.

Study	Model/sex/age or body mass	Intervention (dose; route; timing; duration)	Comparator	Outcomes assessed	Motor ability
(Lee et al., 2020)	MCAO; male mice; 18–20 months	Streptomycin pretreatment, post-MCAO days 1–2; *B. longum* 1 × 10^7^ + *C. symbiosum* 5 × 10^6^ + *F. prausnitzii* 1 × 10^6^ + *L. fermentum* 1 × 10^9^ in 200 μL medium, with inulin at 1 mL/day (10 mg/mL; equivalent to a calculated dose of 10 mg/day); administered by oral gavage once daily on post-MCAO days 3 and 4.	Vehicle medium, inulin alone, or SCFA-producing bacteria alone	I↑, II↑, V↑, VI↓;	↑
(Han et al., 2024)	dMCAO + tCCA occl.; C57BL/6; male; 8–12 weeks	Bifico 105 mg/day; each 210 mg capsule ≥ 1×10^7^ CFU of *Enterococcus faecalis*, *Lactobacillus acidophilus*, *Bifidobacterium longum*; gavage; pre-stroke 28 d; continued post-stroke to day 28 (≈4 weeks)	0.9% NaCl gavage	II↑, III↓,	↑
(Wang et al., 2025)	pMCAO; C57BL/6; male; 6–8 weeks	*Limosilactobacillus reuteri* GMNL-89 8.2×10^7^ or 8.2×10^8^ CFU/kg, *Lacticaseibacillus paracasei* GMNL-133 8.2×10^7^ or 8.2×10^8^ CFU/kg, or G89 + G133 (1:1; 4.1×10^7^–4.1×10^8^ CFU/kg each); gavage; 4 doses pre-stroke, 3 h post-stroke, then qd ×6 d (11 administrations total); to day 7 (≈1 week)	Vehicle (sterile water) gavage; sham for reference	I↑, II↑, III↓, IV↑, VI↓	↑
(Sadler et al., 2020)	PT; C57BL/6J; male; 6–8 weeks	SCFAs in drinking water: acetate 67.5 mM; propionate 25.9 mM; butyrate 40 mM; oral, ad libitum; pre-stroke 4 weeks; continued post-stroke to day 56 (≈8 weeks)	Salt-matched NaCl water (133.4 mM), ad libitum	I↑, II↑, VI↓	↑
(Daniele et al., 2023)	ET-1 sensorimotor cortex stroke; C57BL/6J mice; male; 5–6 weeks	Cerebiome^®^ (*Bifidobacterium longum* R0175 + *Lactobacillus helveticus* R0052), 1×10^9^ CFU/day; oral gavage; post-stroke days 6–20; duration, 14 d	Vehicle gavage; additional sham-vehicle arm	I↑	↑
(Wei et al., 2024)	HAS model (PO 750 mg/kg × 7 d → MCAO I/R); male SD rats, 210–230 g; age not specified	*Parabacteroides distasonis* BNCC 354,946 2×10^8^ CFU/day; gavage; post-stroke days 1–7; once daily	Physiological saline gavage; sham for reference	I↑, II↑, III↓, IV↑, VI↓	↑

MCAO, middle cerebral artery occlusion; dMCAO, distal middle cerebral artery occlusion; pMCAO, permanent middle cerebral artery occlusion; PT, photothrombotic stroke; ET-1, endothelin-1; tCCA occl., transient common carotid artery occlusion; HAS, hyperuricemia-associated stroke; I/R, ischemia–reperfusion; SCFA, short-chain fatty acid; Abx, antibiotics pre-conditioning; CFU, colony-forming units; NaCl, sodium chloride; PO, potassium oxonate; SD, Sprague–Dawley; C57BL/6, C57BL/6 mouse strain; C57BL/6J, C57BL/6J mouse strain; d, day; w, week; qd, once daily. Arrows: ↑ improved; ↓ decreased. Outcome codes: I, gut microbiota composition and SCFA profile; II, neurological function; III, cerebral infarct volume/surrogates; IV, visceral function; V, depressive/affective symptoms; VI, neuroinflammation indices. For Lee et al., the inulin regimen was not specified in the main article but was reported in the Online Data Supplement as 1 mL/day of a 10 mg/mL solution, corresponding to a calculated daily dose of 10 mg/day. For Wei et al., animal age was not reported; therefore, age was not estimated, and the reported body mass of 210–230 g is provided instead.

Despite heterogeneity in strains and schedules, results converged on multi-domain benefits: restoration of gut microbiota composition and SCFA profiles, attenuation of neuroinflammation with BBB-relevant signatures, improvements in neurological function and visceral/metabolic surrogates, and—in several studies—reduced infarct burden alongside better motor behavior (Wang et al., 2025). Mechanistically, these benefits likely involve reinforcement of epithelial and vascular barriers, rebalancing of SCFA-linked signaling, and modulation of microglial and peripheral immune responses. Collectively, these preclinical findings identify candidate mechanisms, formulations, and pharmacodynamic biomarkers for further evaluation, but they do not establish clinically transferable strains, doses, administration routes, or treatment schedules. Translation therefore requires staged human studies that first establish delivery feasibility, biological target engagement, and safety before testing efficacy in combination with standardized exercise-based rehabilitation.

To date, clinical probiotic-based studies in stroke have mainly focused on enteral nutrition–related endpoints such as infections, gastrointestinal complications, length of stay, and nutritional or barrier-associated indices, while neurological assessment has often relied on global scales rather than prespecified motor-specific outcomes (Chen et al., 2022). In contrast, preclinical studies more frequently include behavioral testing, but motor readouts are commonly interpreted alongside infarct size and inflammatory surrogates, and the broader recovery literature continues to highlight short follow-up and insufficiently powered designs as recurrent limitations for modeling true recovery trajectories (Balkaya and Cho, 2019). Accordingly, rehabilitation-oriented trials should prespecify standardized motor outcomes and quantify exercise dose when testing combined exercise and microbiota-targeted strategies, an approach that is now becoming feasible in early clinical work (Wolf and Ergul, 2021).

Incomplete reporting of formulation strength, substrate dose, and participant or animal age in some primary studies precludes quantitative dose–response comparisons. Accordingly, **Tables 1** and **2** should be interpreted as qualitative evidence maps for identifying research priorities rather than as a basis for specifying clinically validated intervention regimens.

Preclinical evidence suggests that combining exercise with SCFA supplementation enhances synaptic density in motor cortical regions (Maejima et al., 2023; Sadler et al., 2020). Beyond serving as substrates, SCFAs act as signaling molecules through receptors including FFAR3 (formerly GPR41), FFAR2 (formerly GPR43), and GPR109A on microglia and astrocytes, and can engage cAMP response element-binding protein–BDNF (CREB–BDNF) signaling and related plasticity programs. Butyrate also functions as a histone deacetylase inhibitor, a mechanism that has been leveraged experimentally to augment training-evoked synaptic protein expression in motor cortical regions and improve motor outcomes after stroke (Maejima et al., 2023). These pathways provide a direct molecular bridge from gut metabolite restoration to M1 reorganization (Qian et al., 2022). In metabolic disease models, combining exercise with butyrate supplementation reshapes gut microbial composition, increases SCFA availability, and improves high-fat-diet-induced metabolic dysfunction, partly through upregulation of hepatic Sestrin 2 and CREB-regulated transcription coactivator 2 signaling (Spychala et al., 2018). Because post-stroke gut microbial communities shift rapidly within days and continue to evolve across recovery phases, intervention timing should be treated as a prespecified design variable in staged microbiota-oriented rehabilitation studies (Jeon et al., 2020). During early stroke recovery, reduced microbial diversity and expansion of pathobionts such as Enterobacteriaceae are linked to heightened systemic inflammation and worse brain injury, supporting early priorities of barrier protection and inflammation control (Peh et al., 2022). Emerging preclinical evidence suggests that prebiotic strategies such as lactulose can reduce endotoxin burden and downregulate TLR4-related inflammatory signaling after ischemic stroke (Yuan et al., 2021). In clinical cohorts, circulating endotoxin and permeability markers, including zonulin, increase after acute ischemic stroke and show associations with clinical outcomes, motivating trials that track these biomarkers alongside function-focused endpoints (Zhong et al., 2022).

## 4 Proposed phase-stratified framework for exercise

We propose this phase-stratified framework to guide hypothesis-driven trial design and biomarker selection, emphasizing the need for validation in adequately powered, stage-specific randomized controlled trials. The microbiota components of this framework represent testable clinical research priorities rather than direct translations of rodent formulations or recommendations for routine care.

These insights inform a phase-specific framework integrating exercise, microbiota strategies, biomarkers, clinical outcomes, and safety considerations. **Table 3** outlines phase-specific exercise prescriptions, microbiota–diet priorities, biomarkers, clinical outcomes, and safety criteria across the acute (days 0–7), subacute (days 8–90), and chronic (>90 days) phases. Safety thresholds can be anchored to established exercise testing and training standards and stroke-specific exercise recommendations, together with dose–response evidence from very early mobilization trials (Bernhardt et al., 2016; Fletcher et al., 2013). Dose–response analyses discourage high total early mobilization doses and suggest that shorter, more frequent sessions may be preferable when titrated to medical stability and patient tolerance (Bernhardt et al., 2016).

**Table 3:** Proposed phase-stratified research framework for exercise–microbiota co-intervention trials after stroke.

Phase and time window	Minimum exercise prescription	Microbiota/diet strategy and delivery considerations	Priority biomarkers and timing	Priority outcomes	Safety and stop rules
Acute phase (days 0–7)	One to three short bouts per day; ratings of perceived exertion 9 to 11; five to ten minutes per bout; bed to chair transitions, sitting balance, assisted stepping	Support nutrition and barrier function; after swallowing and gastrointestinal assessment, trials may evaluate route-compatible, formulation-specific microbiota interventions without directly extrapolating rodent strains, doses, or gavage schedules.	Fecal short-chain fatty acids; plasma C-reactive protein, interleukin-6, and brain-derived neurotrophic factor; baseline and day seven	National Institutes of Health Stroke Scale; early sitting, standing, and walking time; Barthel Index	Defer at rest if systolic pressure is above 200 mmHg or diastolic pressure above 110; stop during exercise if systolic BP ≥ 250 mmHg or diastolic BP ≥ 115 mmHg, or if systolic pressure falls more than 10 with rising workload or if arrhythmia, chest pain, or SpO_2_ < 88% occurs; avoid a high early total dose
Subacute phase (days 8–90)	At least three to five days per week; ratings of perceived exertion 11 to 13 or about 40%–59% heart-rate reserve; twenty to forty minutes per day; add multi-joint resistance at low loads	Build butyrogenic capacity; maintain a fiber-forward diet; consider probiotic and prebiotic combinations when tolerated	Short-chain fatty acids; exploratory 16S or metagenomics; brain-derived neurotrophic factor; baseline and weeks four to eight	Fugl-Meyer upper and lower extremity, Action Research Arm Test, 10-meter gait speed, six-minute walk test, Berg Balance Scale with prespecified time points	Same thresholds as the acute phase with attention to orthostatic symptoms and fatigue
Chronic phase (> 90 days)	At least 150–300 min/week of moderate-intensity aerobic exercise; two to three days per week of progressive resistance training; integrate task-specific practice	Maintain Mediterranean-style dietary pattern with high fiber intake; continue individualized microbiota strategies if prior response is favorable	Short-chain fatty acids, brain-derived neurotrophic factor, and inflammatory panel at periodic follow-up	Core set as above and include longer-term events such as rehospitalization where appropriate	Apply the same thresholds and align with long-term secondary prevention targets

### 4.1 Acute phase (days 0–7)

The acute-phase goal is to stabilize hemodynamics, preserve gut barrier function, and prevent deconditioning. Light mobilization should begin early and be titrated to tolerance. See **Table 3** for phase-specific targets. Key activities include bed-to-chair transfers, sitting balance, and assisted ambulation, while avoiding breath-holding. Microbiota-oriented interventions during the acute phase should remain investigational. Following formal swallowing, nutritional, and gastrointestinal-tolerance assessment, trials may evaluate formulation-specific prebiotic, probiotic, or postbiotic strategies delivered through a clinically appropriate oral or enteral route. Participants should be stratified by feeding route and gastrointestinal motility. Exposure measures should be formulation-specific: viable dose and strain-specific fecal recovery for live microbial products; substrate dose and metabolite response for prebiotics; and bioactive composition and dose for postbiotics. Treatment adherence, gastrointestinal tolerance, and adverse events should also be recorded. No rodent-derived strain, dose, or gavage schedule should be directly extrapolated to clinical care without staged feasibility and safety evaluation. Biomarker collection focuses on fecal SCFAs by gas chromatography mass spectrometry where feasible and plasma C-reactive protein, interleukin-6, and brain-derived neurotrophic factor at baseline and day seven. In trials, record early functional exposure as time spent sitting, standing, and walking. Defer exercise if resting systolic blood pressure (BP) is ≥ 200 mmHg or diastolic BP is ≥ 110 mmHg. Exercise should be terminated if systolic BP is ≥ 250 mmHg or diastolic BP is ≥ 115 mmHg, if systolic BP falls by > 10 mmHg with increasing workload, if arrhythmia or chest pain occurs, or if peripheral oxygen saturation (SpO_2_) falls below 88% (Winstein et al., 2016). Avoid high total doses of very-early mobilization; prefer short, frequent bouts individualized to tolerance.

### 4.2 Subacute phase (days 8–90)

During the subacute phase, the therapeutic objective shifts toward maximizing neuroplastic potential while establishing metabolic flexibility. This involves a regimen of aerobic exercise titrated to patient tolerance, complemented by nutritional interventions designed to optimize the gut-brain metabolic axis. For trials, apply a light-to-moderate aerobic stimulus three to five days per week for 20 to 40 minutes, with progressive low-load multi-joint resistance work; adapt dosing to tolerance and predefined safety thresholds. A fiber-rich diet is maintained, with probiotic or prebiotic combinations introduced as tolerated to promote butyrate-producing taxa. Biomarker panels may include exploratory 16S or metagenomic profiling, while SCFAs and BDNF remain prioritized at baseline and weeks 4–8. Trials may prioritize Fugl–Meyer upper and lower extremity scores, the Action Research Arm Test, 10-meter gait speed, the six-minute walk test, and the Berg Balance Scale at prespecified time points aligned with consensus recommendations. Blood pressure–based stop criteria and monitoring for orthostatic symptoms and fatigue remain essential.

### 4.3 Chronic phase (> 90 days)

The focus is consolidation of neuroplastic gains and reduction of cardiometabolic risk. For trials, target 150 to 300 minutes per week of moderate aerobic exercise plus two to three days of progressive resistance training, integrated with task-specific practice and individualized to goals and tolerance. A high-fiber Mediterranean-style diet is maintained. Microbiota-targeted strategies continue if previous responses suggest benefit. SCFAs, BDNF, and inflammatory markers may be monitored at extended intervals. The same core outcomes are retained, with addition of longer-term events such as rehospitalization where relevant, and targets are aligned with recommendations for stroke survivors in long-term prevention.

This framework is investigational; implementation in routine care should await validation in adequately powered, randomized, and phase-specific clinical trials.

## 5 Challenges and future directions

### 5.1 Preclinical-to-clinical translation remains a major limitation of the proposed framework

Rodent stroke models are useful for establishing mechanistic plausibility, but they cannot directly determine clinically appropriate microbial strains, doses, delivery routes, or treatment schedules. Species-specific differences in baseline microbial ecology, diet, immune physiology, gastrointestinal anatomy, and colonization resistance may alter the survival and functional activity of candidate strains. Moreover, orogastric gavage provides a standardized bolus exposure and bypasses palatability, voluntary intake, adherence, and oropharyngeal swallowing, whereas microbiota-directed interventions in stroke survivors must be delivered through oral preparations, texture-modified foods, nasogastric tubes, or gastrostomy according to swallowing safety and nutritional status. This distinction is particularly relevant because post-stroke dysphagia and impaired upper gastrointestinal motility may coexist, and greater dysphagia severity has been associated with increased gastric residual volumes and delayed gastric emptying in neurocritical care populations (Muhle et al., 2021). Stroke-related autonomic dysfunction, immobility, enteral feeding, and exposure to antibiotics, proton-pump inhibitors, laxatives, and other medications may further alter intestinal transit and colonization dynamics. Accordingly, animal-to-human dose conversion alone is insufficient. Early-phase trials should stratify participants by swallowing status, feeding route, gastrointestinal motility, and recovery phase; use clinically scalable and route-compatible formulations; and apply formulation-specific exposure measures, including viable dose and strain-specific fecal recovery for live microbial products, substrate dose and metabolite response for prebiotics, and bioactive composition and dose for postbiotics, alongside gastrointestinal tolerance and adverse-event monitoring, before evaluating effects on motor recovery.

Translational progress is constrained by the lack of predictive models that capture interindividual variability in microbiota responses to exercise and microbiota-directed therapies (Jeon et al., 2020). A practical next step is to integrate metagenomic sequencing with longitudinal multi-omics and digital phenotyping, including ingestible gastrointestinal devices capable of sensing luminal physiology such as pH and emerging wearable platforms for continuous metabolite monitoring (Thwaites et al., 2024). Mechanistic priorities are likely phase-dependent: early after stroke, barrier disruption and systemic inflammation dominate, whereas later recovery increasingly depends on neural repair and plasticity, supporting time-resolved sampling across recovery stages (Irisa and Shichita, 2025). Exercise appears to act as a hormetic stressor on the gut ecosystem, with moderate training generally associated with more favorable microbiome profiles, while excessive or very high-intensity training is linked to increased gut permeability and inflammatory responses (Allen et al., 2018). Accordingly, post-stroke trials should prespecify exercise dose and avoid high early total doses, because dose–response analyses in very early mobilization discourage long-duration early sessions and favor short, frequent bouts individualized to tolerance (Bernhardt et al., 2016). For microbiota-based strategies, development frameworks for live biotherapeutic products emphasize defining colonization dynamics and pharmacodynamic biomarkers to support rational dose selection and to reduce the risk of both under- and over-exposure. Preclinical stroke data further suggest that single-component approaches such as inulin alone or SCFA-producing bacteria alone may be insufficient, whereas combining a prebiotic substrate with SCFA producers can more robustly improve neurological and behavioral outcomes, supporting combination designs in staged rehabilitation studies (Fang et al., 2023; Lee et al., 2020). Future directions include engineered live biotherapeutics and monitoring-enabled precision rehabilitation programs to enable microbiota stratification and exercise dose matching. We further propose that a eubiotic microbiome may provide tonic vagal afferent drive that primes motor learning during task practice, conceptually resembling an intrinsic form of VNS that raises neuromodulatory readiness for consolidation. This hypothesis is testable by coupling longitudinal microbiome metabolomics with autonomic readouts such as heart rate variability and with behavioral retention metrics for motor skills across rehabilitation phases (Lai et al., 2024).

## 6 Conclusion

The limited efficacy of unimodal stroke rehabilitation has driven growing interest in integrative strategies targeting systemic recovery pathways. Emerging preclinical evidence identifies the gut microbiota as a modifiable target that may enhance motor recovery via metabolic and immune modulation. Aligning microbiota-based interventions with phase-specific recovery biology may inform the design of individualized rehabilitation protocols.

Combining structured exercise with microbiota-directed strategies may support motor and systemic recovery; however, current evidence remains limited to preclinical models and early, formulation-specific clinical studies. The superiority of combined interventions over single-modality approaches and the contribution of microbiota-mediated mechanisms require confirmation in adequately powered, phase-specific randomized controlled trials.

The proposed phase-stratified framework provides a structured approach for testing combined exercise and microbiota-directed interventions. Its clinical utility and any interaction between the two components require confirmation in adequately powered, phase-specific randomized trials.
